# Surveilling brain damage using brain biomarkers in hypoglycemic neonatal calves with diarrhea

**DOI:** 10.3389/fvets.2023.1240846

**Published:** 2023-10-31

**Authors:** Merve Ider, Amir Naseri, Mahmut Ok, Alper Erturk, Murat Kaan Durgut, Suleyman Serhat Iyigun

**Affiliations:** ^1^Faculty of Veterinary Medicine, Department of Internal Medicine, Selcuk University, Konya, Türkiye; ^2^Faculty of Veterinary Medicine, Department of Internal Medicine, Hatay Mustafa Kemal University, Hatay, Türkiye

**Keywords:** hypoglycemia, neonatal calf, brain injury, biomarkers, prognosis

## Abstract

Hypoglycemia is a condition associated with neonatal diarrhea in calves, leading to increased mortality and neurological clinical signs. The aim of the present study was to determine the development of brain damage in hypoglycemic calves with neonatal diarrhea and the diagnostic and prognostic significance of these biomarkers. Ten healthy and 50 hypoglycemic calves with diarrhea were included in the study. Clinical examination, blood gases and complete blood count were performed at admission. Blood serum calcium-binding protein B (S100B), neuron-specific enolase (NSE), glial fibrillary acidic protein (GFAP), ubiquitin carboxyl-terminal hydrolysis isoenzyme-1 (UCHL-1), activitin A (ACT), adrenomodullin (AM) concentrations, and creatine kinase-BB (CK-BB) enzyme activity were measured using commercial bovine-specific ELISA kits to assess brain damage. Of the hypoglycemic calves enrolled in the study, 13 (26%) survived and 37 (74%) died. In addition, 32 (64%) of the calves had severe acidosis and 24 (48%) had sepsis. S100B, GFAP, UCHL-1, CK-BB (*p* < 0.001) and NSE (*p* < 0.05) concentrations were significantly higher in hypoglycemic calves compared to healthy calves, while ACT concentrations were lower. Blood glucose concentration was negatively correlated with serum S100B, GFAP, UCHL-1, and CK-BB enzyme activity and positively correlated with ACT in hypoglycemic calves (*p* < 0.01). Brain injury biomarkers were not predictive of mortality (*p* > 0.05). Morever, severe hypoglycemia, severe acidosis and sepsis variables were not found to have sufficient capacity to predict mortality when considered alone or together (*p* > 0.05). In conclusion, brain damage may develop as a consequence of hypoglycemia in calves. S100B, NSE, GFAP, UCHL-1, ACT, and CK-BB concentrations can be used to diagnose brain damage in hypoglycemic calves. However, the variables of severe hypoglycemia, severe acidosis, and sepsis together with the biomarkers of brain injury have a limited value in predicting the prognosis of neonatal calves with diarrhea.

## 1. Introduction

Neonatal diarrhea remains an important herd health problem in suckling calves, which can lead to mortality and economic losses (1). Hypoglycemia, azotemia, hyponatremia, hyperkalemia, septicemia, hyperlactatemia, and strong ion (metabolic) acidosis are the most common laboratory complications in calves with diarrhea (2, 3). Among these complications, hypoglycemia has been reported to occur in a very small percentage of cases of acute diarrhea and is associated with increased mortality (4).

Under physiological conditions, glucose is the primary energy substrate for the brain in both animals and humans. Decreased plasma glucose levels for various reasons impair brain glucose metabolism, resulting in functional brain damage (5). In addition, hypoglycemia can cause dysfunction of blood–brain barrier (BBB) permeability (6) and structural and functional disturbances in the peripheral nervous system (5). Therefore, it has been reported that the risk of permanent brain damage in hypoglycemic neonates is high (7, 8).

Recently, brain injury biomarker concentrations have been used in the diagnosis and prognosis of various central nervous system (CNS) disorders (9–12). Among these biomarkers, calcium-binding protein B (S100B) and neuron-specific enolase (NSE) have been reported to have increased concentrations in hypoglycemic newborns in association with the development of brain injury (8). Concentration of glial fibrillary acidic protein (GFAP), an intermediate cytoskeletal filament protein specific for astrocytes, have been found to increase as a result of glial damage during abnormal glucose homeostasis (11, 13). Ubiquitin C-terminal hydrolase-1 (UCHL-1) is a reliable biomarker that is widely expressed in neurons and neuroendocrine cells (14). Clinical studies in calves (12) have reported that UCHL-1 is a useful biomarker for the detection of hypoxic–ischemic encephalopathy. Activin A (ACT) protein has an important biological effect on neuronal cell differentiation (15). It has been found that ACT concentrations increase after neuronal damage related to oxygen–glucose deprivation, and exogenous administration of ACT has a neuroprotective effect by preventing apoptosis in neurons (10). Adrenomodullin (AM), a hypotensive vasodilator peptide, is synthesized in the organism as preproadrenomodulin. Studies in the rat model showed that oxygen and glucose deprivation increased AM in several brain regions compared to control animals (9). Creatine kinase-BB (CK-BB) is an isoenzyme that is found in astrocytes (16), and it has been reported that the activity of the CK-BB enzyme is significantly increased in infants with neurological disorders (17).

Although our knowledge of the brain damage caused by hypoglycemia in human medicine is now well advanced, the studies in veterinary medicine are still limited. The present study was designed with the hypothesis that hypoglycemia may lead to brain damage in neonatal calves. The aim of this study was to evaluate brain damage in hypoglycemic calves using brain-specific biomarkers and to determine their diagnostic and prognostic significance.

## 2. Materials and methods

The study was conducted between January 2022 and March 2023 at the Department of Internal Medicine, Faculty of Veterinary Medicine, Selcuk University, Konya, Türkiye. The study protocol was approved by the Institutional Ethics Committee of the Faculty of Veterinary Medicine, Selcuk University (No. 2022/07).

### 2.1. Study groups

Ten healthy calves (8 Holstein and 2 Simmental), > 280 days gestation, 2–14 days old, were enrolled in the study as a control group. Calves were considered healthy based on clinical examination and laboratory findings (18). Calves were born naturally at the faculty farm. Calves with dystocia, prematurity, congenital abnormalities, hypoglycemia, acidemia, and suspected infection were excluded from the study.

Fifty calves with diarrhea (26 Holstein, 12 Simmental, 7 Brown Swiss, and 5 Charolais), > 280 days gestation, 2–14 days old were enrolled in the study as the hypoglycemic group. All calves were hospitalized for 3 days and received standard care and a feeding protocol after admission to the neonatal intensive care unit (19). Criteria for hypoglycemia were defined as a blood glucose concentration < 79.2 mg/dL. Mild hypoglycemia and severe hypoglycemia were also defined as blood glucose concentrations between 36 and 79 mg/dL and < 36 mg/dL, respectively (20). In the first step, the results of the study (blood gas analysis, CBC and brain related biomarkers) were compared between healthy (*n* = 10) and hypoglycemic (*n* = 50) calves. The hypoglycemic calves were then divided into mild hypoglycemic (*n* = 8) and severe hypoglycemic groups (*n* = 42) and the concentrations of brain related biomarkers were compared. Next, brain-related biomarker concentrations were compared between surviving (*n* = 13) and non-surviving (*n* = 37) hypoglycemic calves. Finally, calves with sepsis, severe acidosis (pH < 7.20) (21), and severe hypoglycemia were adjusted to find a model for mortality. Sepsis was described as the existence of systemic inflammatory response syndrome (SIRS) and a suspected or proven infection. Definitions for SIRS were based on the presence of the two or more of the following abnormalities: leukocyte count (leukocytosis or leukopenia, or band neutrophils >10%), abnormal rectal temperature, tachycardia, and tachypnea (22).

### 2.2. Clinical examination

All clinical examinations followed a standardized protocol and were performed by the same investigators (MI and AE) on admission and during hospitalization. Degree of enophthalmos (none, mild to moderate, severe), mental status (alert, depressed, comatose), suckling reflex (strong, weak, absent), and posture (standing, sternal, lateral recumbency) were assessed. Heart rate (beats/min), rectal temperature (°C), respiratory rate (breaths/min), mucous membranes (hyperemic or cyanotic), and capillary refill time were also recorded (4, 19).

### 2.3. Collection of blood samples

Blood samples were collected from the calves at the time of admission. Blood samples for blood gas analysis, complete blood count (CBC), and biomarkers of brain injury were collected from the jugular vein. For blood gas measurements, plastic syringes containing sodium heparin were used. Tubes containing K_3_EDTA were used to analyze the CBC. Blood gas and CBC measurements were performed within 5 to 10 min after the sample was collected. Non-anticoagulant tubes were used for serum collection. Blood samples collected for biomarker analysis were kept at room temperature for 15 min and then centrifuged at 20 × *g* for 10 min. Sera were collected and stored at −80°C.

#### 2.3.1. Blood gas analysis

Venous blood pH, partial carbon dioxide pressure (pCO_2_), partial oxygen pressure (pO_2_), oxygen saturation (SO_2_), potassium (K), sodium (Na), calcium (Ca), chlorine (Cl), glucose (Glu), lactate (Lac), base deficit (BE), and bicarbonate (HCO_3_) were measured using an automated blood gas analyzer (ABL 90 Flex, Radiometer, Brea, CA, United States).

#### 2.3.2. Complete blood count (CBC) analysis

Total leukocytes (WBC), lymphocytes (Lym), monocytes (Mon), granulocytes (Gra), erythrocytes (RBC), hematocrit (HCT), hemoglobin (Hb), and platelets (PLT) were measured using an automated cell counter (MS4e, Melet Schlosing Laboratories, Osny, France).

#### 2.3.3. Evaluation of brain-related biomarkers

Serum S100B, UCHL-1, (Bioassay Technology Laboratory, Shanghai, China), NSE, GFAP, ACT, AM (MyBioSource, San Diego, CA, United States), and CK-BB (ELK Biotechnology Co., Ltd., Wuhan, China) concentrations were measured using commercial bovine-specific ELISA test kits according to the manufacturer’s instructions. Bovine S100B commercial ELISA kit (Bioassay Technology Laboratory, Shanghai, China, Lot: 202110012), bovine NSE commercial ELISA kit (MyBioSource®, San Diego, CA, United States, Lot: 36379821), bovine GFAP commercial sandwich ELISA kit (MyBioSource®, San Diego, CA, United States, Lot: 34358721), bovine UCHL-1 commercial ELISA kit (Bioassay Technology Laboratory, Shanghai, China, Lot: 202110012), bovine ACT commercial ELISA kit (MyBioSource®, San Diego, CA, United States, Lot: 20211022C), bovine AM commercial ELISA kit (MyBioSource®, San Diego, CA, United States, Lot: 38400921), and bovine CK-BB commercial ELISA kit (ELK Biotechnology, Wuhan, China, Lot: 20330054610) were used for biomarker ELISA analyses. The intra-assay coefficient of variation (CV), inter-assay CV, and minimum detectable concentrations (MDC) for biomarkers were ≤ 8%, ≤ 10%, and 0. 26 ng/mL for S100B, ≤ 8%, ≤ 12% and > 0.06 ng/mL for NSE, ≤ 8%, ≤ 12% and > 0.06 ng/mL for GFAP, ≤ 8%, ≤ 10%, and 35.7 ng/mL for UCHL-1, < 10%, < 10% and 1.0 pg/mL for ACT, ≤8%, ≤12% and 5 pg/mL for AM, and < 8%, < 10% and 0.59 ng/mL for CK-BB, respectively.

### 2.4. Statistical analysis

#### 2.4.1. Power analysis

The 95% confidence interval (CI) and effect size (margin of error) for the hypoglycemic calves with diarrhea were included in the calculation. Previous study in neonatal calves with asphyxia has demonstrated brain damage in 50% calves (12). Based on this assumption, 50 neonatal calves were considered necessary to identify brain damage associated with hypoglycemia with 80% power and 5% alpha error level using a 2-tailed test.

#### 2.4.2. Analysis of variances

The SPSS 25 statistical program (IBM Corp®, 2017, Armonk, NY, United States) was used to evaluate the data. The Kolmogorov–Smirnov test was used to determine normality of variables and homogeneity of variances. Parametric data were expressed as mean ± SD and evaluated by Student’s t-test. Non-parametric data were expressed as median (minimum/maximum) and evaluated using the Mann–Whitney U test. The Spearman correlation test was used to determine the correlation between variables. Binary logistic regression was used to evaluate the association of severe hypoglycemia, severe acidemia, and sepsis with mortality. The goodness of fit of the model was assessed using Pearson chi-squared. Receiver operating characteristic (ROC) analysis was performed to determine the prognostic cut-off, sensitivity, and specificity of the variables in non-surviving and surviving hypoglycemic calves. In addition, the same test was used to evaluate the ability of severe hypoglycemia, severe acidosis, and sepsis to predict mortality. Statistical significance was considered as *p* < 0.05.

## 3. Results

### 3.1. Clinical findings

Fifty hypoglycemic calves with diarrhea and 10 healthy calves were included in the study. The mean body weight of the calves was 42.37 ± 2.79 in the hypoglycemic group and 44.54 ± 2.35 in the healthy group. The most prominent clinical signs in hypoglycemic calves were hypothermia, lethargy, lateral recumbency, loss of suckling reflex, severe depression, and coma. CNS-related symptoms such as convulsions, opisthotonos, and nystagmus were seen in 6 (12%) hypoglycemic calves. Of the hypoglycemic calves, 37 (74%) did not survive and 13 (26%) survived. It was determined that 42 (84%) of the hypoglycemic calves had severe hypoglycemia. In addition, 32 (64%) and 24 (48%) calves had severe acidosis and sepsis, respectively.

### 3.2. Blood gas and CBC analysis

At the time of admission, pH, pO_2_, SO_2_, glucose, BE, and HCO_3_ levels of hypoglycemic calves were significantly lower and pCO_2_, lactate, and K levels were higher than healthy calves (*p* < 0.05). Total leukocytes, Lym, Mon, RBC, Hb, and PLT levels of hypoglycemic calves were significantly higher than healthy calves (*p* < 0.05) (Table 1).

**Table 1:** Venous blood gas and CBC parameters of healthy and hypoglycemic calves.

Variable	Study groups		*p* value
	Healthy calves	Hypoglycemic calves	
pH	7.41 ± 0.03	7.11 ± 0.16	< 0.001
pCO_2_ (mmHg)	39.00 ± 7.60	56.50 ± 14.12	< 0.001
pO_2_ (mmHg)	49.20 (23.40–97.10)	22.05 (12.40–62.50)	< 0.001
SO_2_ (%)	96.20 (58.90–101.30)	38.10 (6.00–89.50)	< 0.001
K (mmol/L)	4.46 ± 0.30	5.19 ± 1.11	< 0.001
Na (mmol/L)	146.10 ± 4.17	146.42 ± 8.60	0.860
Ca (mmol/L)	0.96 ± 0.19	1.02 ± 0.18	0.425
Cl (mmol/L)	104.40 ± 4.55	101.32 ± 9.07	0.122
Glu (mg/dL)	103.00 (82.00–137.00)	15.00 (1.00–55.00)	< 0.001
Lac (mmol/L)	4.00 (2.60–5.20)	7.95 (0.10–24.00)	0.027
BE (mmol/L)	0.25 (−7.20–5.40)	−8.95 (−26.20–6.50)	< 0.001
HCO_3_ (mmol/L)	24.96 ± 3.66	15.97 ± 5.87	< 0.001
WBC (cells/mL)	7.95 (5.25–11.33)	13.13 (3.99–76.97)	0.005
Lym (cells/mL)	2.27 (1.70–3.53)	5.58 (1.82–72.89)	< 0.001
Mon (cells/mL)	0.34 (0.18–0.84)	0.52 (0.09–4.74)	0.039
Gra (cells/mL)	4.76 (1.39–9.26)	5.43 (0.50–50.33)	0.539
RBC (×10^3^ cells/mL)	7.89 ± 0.81	9.39 ± 2.66	0.002
HCT (%)	33.10 ± 8.76	38.43 ± 12.49	0.122
Hb (g/dL)	9.55 ± 1.65	11.85 ± 3.52	0.003
PLT (cells/mL)	195.80 ± 34.40	273.46 ± 186.02	0.008

Partial carbon dioxide pressure (pCO_2_), partial oxygen pressure (pO_2_), oxygen saturation (SO_2_), potassium (K), sodium (Na), calcium (Ca), chlorine (Cl), glucose (Glu), lactate (Lac), base deficit (BE), bicarbonate (HCO_3_), total leukocytes (WBC), lymphocytes (Lym), monocytes (Mon), granulocytes (Gra), erythrocytes (RBC), hematocrit (HCT), hemoglobin (Hb), platelets (PLT).

### 3.3. Brain-related biomarkers analysis

Biomarker concentrations of healthy and hypoglycemic calves are shown in Table 2. S100B, GFAP, UCHL-1, CK-BB (*p* < 0.001) and NSE (*p* < 0.05) concentrations of hypoglycemic calves were significantly higher than the control group. ACT concentrations were significantly lower in hypoglycemic calves compared to healthy calves. There was no significant change in AM concentrations between hypoglycemic and healthy calves (*p* > 0.05) (Table 2). Additionally, no significant difference in brain-related biomarker concentrations was observed between mildly and severely hypoglycemic calves (Table 3).

**Table 2:** Biomarker concentrations findings in healthy and hypoglycemic calves.

Variable	Study groups		*p* value
	Healthy calves	Hypoglycemic calves	
S100B (ng/mL)	13.08 ± 3.51	24.18 ± 4.55	< 0.001
NSE (ng/mL)	3.59 ± 0.91	4.69 ± 2.08	0.013
GFAP (ng/mL)	1.44 ± 0.47	3.21 ± 1.76	< 0.001
UCHL-1 (ng/L)	837.10 ± 152.83	1726.22 ± 411.78	< 0.001
ACT (pg/mL)	6214.16 ± 913.51	3467.96 ± 1570.33	< 0.001
AM (pg/mL)	188.24 ± 76.95	178.91 ± 68.29	0.728
CK-BB (ng/mL)	4.97 ± 1.63	9.59 ± 2.67	< 0.001

Calcium binding protein B (S100B), neuron-specific enolase (NSE), glial fibrillary acidic protein (GFAP), ubiquitin carboxyl terminal hydrolysis isoenzyme-1 (UCHL-1), activitin A (ACT), adrenomodullin (AM), creatine kinase-BB (CK-BB).

**Table 3:** Comparison of brain biomarker concentrations in mild and severe hypoglycemic calves.

Variable	Hypoglycemic calves		*p* value
	Severe hypoglycemia	Mild hypoglycemia	
S100B (ng/mL)	22.72 ± 3.90	24.46 ± 4.66	0.289
NSE (ng/mL)	4.01 ± 1.03	4.82 ± 2.21	0.124
GFAP (ng/mL)	2.99 ± 0.75	3.25 ± 1.89	0.527
UCHL-1 (ng/L)	1668.14 ± 279.63	1737.55 ± 434.72	0.572
ACT (pg/mL)	2892.47 ± 1873.36	3580.25 ± 1505.26	0.354
AM (pg/mL)	162.58 ± 31.23	182.10 ± 73.22	0.231
CK-BB (ng/mL)	9.13 ± 2.66	9.68 ± 2.69	0.606

Calcium binding protein B (S100B), neuron-specific enolase (NSE), glial fibrillary acidic protein (GFAP), ubiquitin carboxyl terminal hydrolysis isoenzyme-1 (UCHL-1), activitin A (ACT), adrenomodullin (AM), creatine kinase-BB (CK-BB).

### 3.4. Correlation analysis

**Figure 1:**
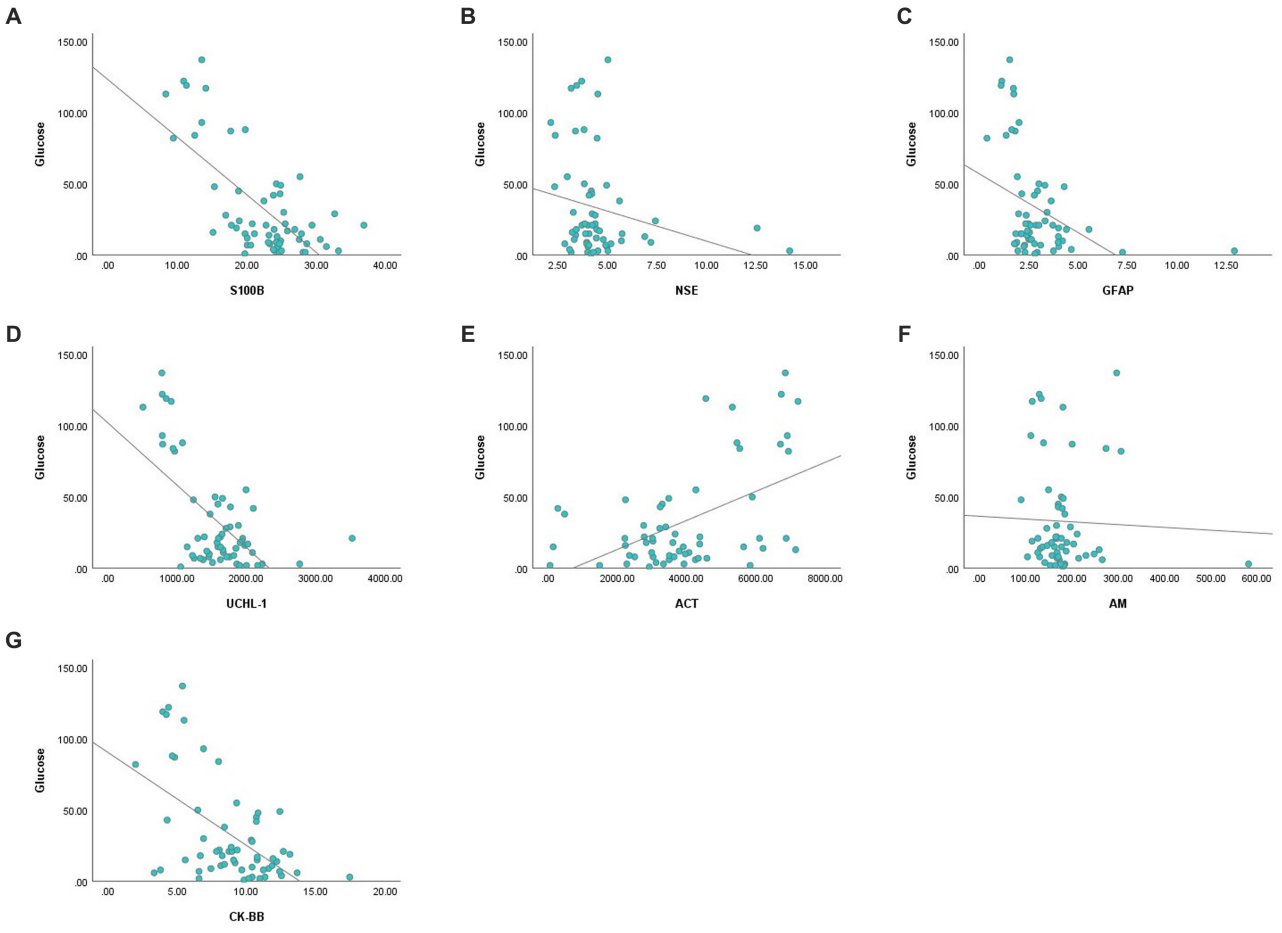
Correlation analysis graphs between blood glucose and serum concentrations of S100B **(A)**, NSE **(B)**, GFAP **(C)**, UCHL-1 **(D)**, ACT **(E)**, AM **(F)**, and CK-BB **(G)**.

There was a negative correlation between blood glucose concentration and serum S100B, GFAP, UCHL-1 concentration and CK-BB enzyme activity, and positive correlation with ACT (*p* < 0.01) (Figure 1). There was no association between glucose concentration and serum AM and NSE.

### 3.5. Prognostic indicators analysis

#### 3.5.1. Brain-related biomarker

**Figure 2:**
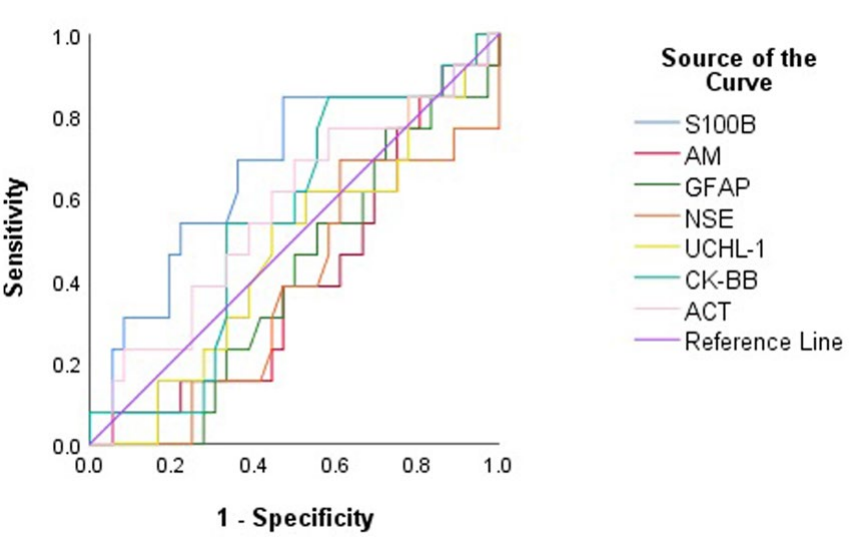
Receiver operating characteristic (ROC) curve analysis to discriminate between surviving and non-surviving hypoglycemic calves based on serum concentrations of brain biomarkers.

None of S100B, NSE, GFAP, UCHL-1, ACT, AM, and CK-BB were found to be significant (*p* > 0.05) in predicting mortality in calves with hypoglycemia (Table 4; Figure 2).

**Table 4:** The area under the curve (AUC), standard error, confidence interval (95%), optimal cut-off values, and corresponding sensitivity and specificity for predicting mortality in non-surviving calves with hypoglycemia.

Variable	AUC	Standard error	*p v*alue	Asymptotic 95% confidence interval		Sensitivity	Specificity	Cut-off value
				Lower band	Upper bound			
S100B (ng/mL)	0.663	0.093	0.083	0.482	0.846	69	64	23.95
NSE (ng/mL)	0.377	0.089	0.193	0.203	0.551	53	42	4.29
GFAP (ng/mL)	0.405	0.086	0.314	0.237	0.573	53	45	2.88
UCHL-1 (ng/L)	0.470	0.091	0.751	0.292	0.648	61	48	1706.47
ACT (pg/mL)	0.571	0.095	0.455	0.384	0.757	61	56	3359.66
AM (pg/mL)	0.400	0.089	0.287	0.226	0.0573	61	51	179.34
CK-BB (ng/mL)	0.548	0.089	0.610	0.373	0.723	61	50	10.24

Calcium binding protein B (S100B), neuron-specific enolase (NSE), glial fibrillary acidic protein (GFAP), ubiquitin carboxyl terminal hydrolysis isoenzyme-1 (UCHL-1), activitin A (ACT), adrenomodullin (AM), creatine kinase-BB (CK-BB).

#### 3.5.2. Logistic regression analysis and capacity of the model

Logistic regression analysis showed that the presence of sepsis, severe acidosis (pH < 7.20), and severe hypoglycemia (glucose <36 mg/dL) were not significantly associated with mortality when each variable was included in the analysis separately (Table 5). Furthermore, when sepsis, severe acidosis, and severe hypoglycemia were considered together, this model was found to be inadequate in predicting mortality (R chi-squared = 2.789, *p* = 0.425).

**Table 5:** Logistic regression for mortality comparing sepsis, severe hypoglycemia, and severe acidosis in hypoglycemic calves.

Variable	B	SE	Wald	df	Sig.	EXP (B)	Confidence interval %95	
							Lower	Upper
Sepsis	0.029	0.667	0.002	1	0.966	1.029	0.278	3.806
Severe hypoglycemia (< 36 mg/dL)	−0.610	0.967	0.398	1	0.528	0.543	0.082	3.617
Severe acidosis (< 7.20)	1.174	0.712	2.718	1	0.099	3.235	0.801	13.063

**Figure 3:**
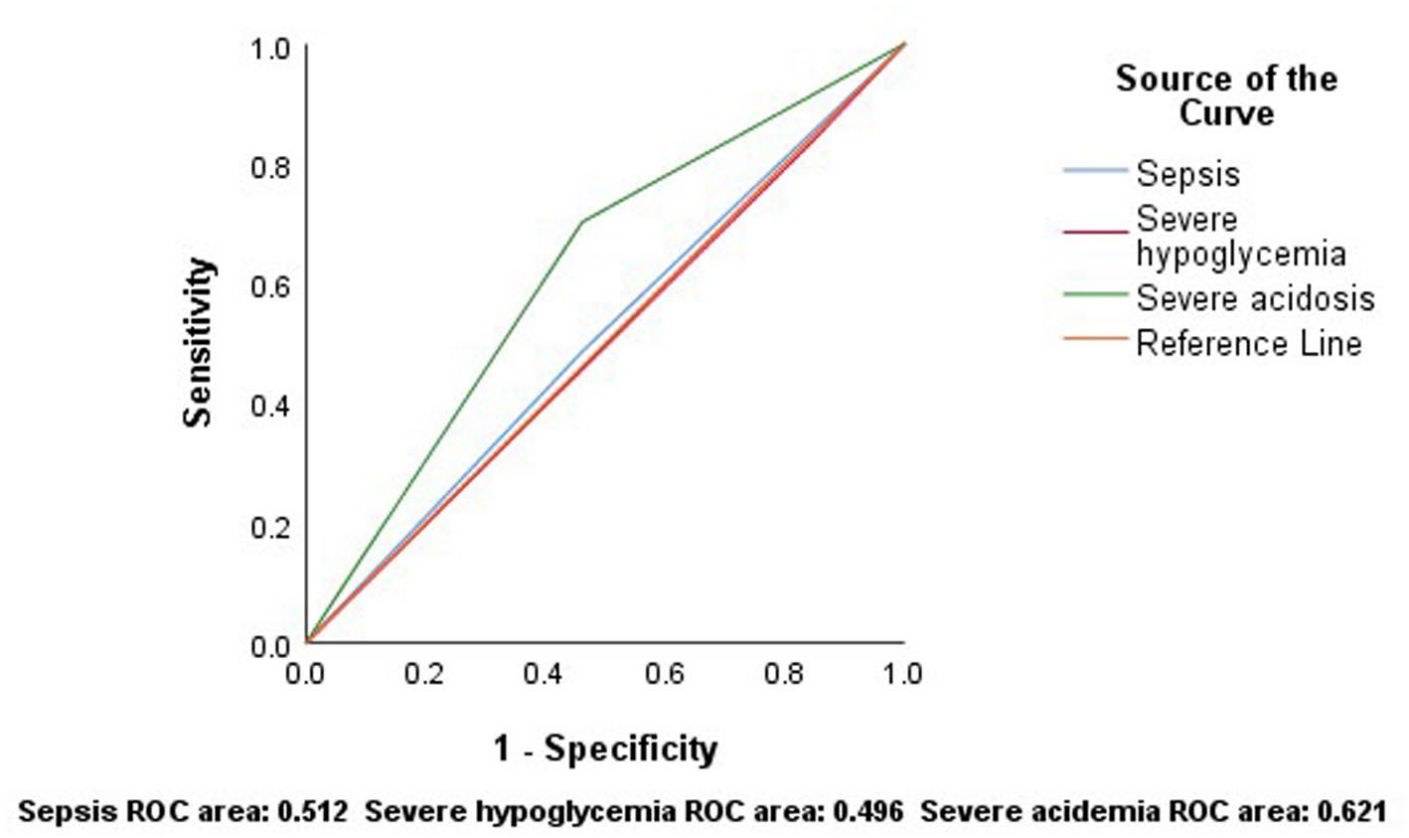
According to the results of ROC analysis, sepsis, severe hypoglycemia and severe acidosis have low sensitivity and specificity in predicting mortality.

The ROC curve for sepsis to predict mortality showed an area under the curve (AUC) (95% CI) of 0.512 (0.328–0.697), with a sensitivity of 48% and a specificity of 54%. For severe hypoglycemia, the AUC (95% CI) was 0.496 (0.312–0.680), with 83% sensitivity and 16% specificity. For severe acidosis, the AUC (95% CI) was 0.621 (0.438–0.803), with a sensitivity of 70% and a specificity of 54% (Figure 3).

## 4. Discussion

In this study, S100B, NSE, GFAP, UCHL-1, ACT, AM concentrations, and CK-BB enzyme activity were measured in blood serum samples from hypoglycemic calves with neonatal diarrhea. Our results showed that significant changes in S100B, NSE, GFAP, UCHL-1, ACT concentrations, and CK-BB enzyme activity occurred in calves with diarrhea related to hypoglycemia, and hypoglycemia was associated with high mortality. However, the biomarkers of brain injury were not useful in the prediction of mortality in calves with hypoglycemia. In addition, severe hypoglycemia, severe acidosis and sepsis variables were found to be insufficient to predict mortality alone or together.

Hypoglycemia in calves is a condition resulting from neonatal diarrhea, endotoxemia and asphyxia and is associated with mortality (4, 19, 23). The poor prognosis of severely hypoglycemic calves is explained by concurrent health problems including diffuse peritonitis, septicemia and acidosis (4). Trefz et al. (4) found that the survival rate was 74.0% for normoglycemic calves and 20.6% for calves with severe hypoglycemia. In the study, the survival rates of calves with plasma glucose concentrations <1 mmol/L and 1–1.9 mmol/L were 9.6 and 26.4%, respectively. In another study, the mortality rate of calves with severe hypoglycemia was reported to be 79.4% (19). In the present study, 26% of 50 hypoglycemic calves survived and 74% died. In addition, severe acidosis was observed in 64% and sepsis in 48% of hypoglycemic calves. The high mortality rate of hypoglycemic calves in our study may be related to malnutrition, sepsis and metabolic acidosis (4).

Hypoglycemia in humans and newborn calves is usually asymptomatic. Similar to previous studies (4, 24, 25), only 12% of enrolled calves had CNS-related clinical signs. The lower incidence of clinical signs related to the CNS in hypoglycemic calves may be explained by the fact that newborn dogs and calves are more tolerant to the deleterious effects of hypoglycemia due to their ability to use L-lactate as a fuel for the brain (26, 27).

Considering the potential adverse effects of hypoglycemia on the brain, this study investigated brain damage biomarkers in hypoglycemic calves with neonatal diarrhea. Because there is limited research on these biomarkers in the veterinary setting, our findings are discussed with the human literature.

Concurrent increases in the concentrations of S100B (released from astrocytes and oligodendrocytes) and NSE (released from neurons) have been interpreted as indicators of brain damage (28, 29). However, data on the relationship between these biomarkers and brain damage due to glucose dysregulation is limited. While serum S100B concentrations do not change in diabetic patients with metabolically impaired BBB (30), elevated S100B and NSE concentrations after severe hypoglycemia have been evaluated as indicators of permanent neurological damage (31). Higher concentrations of S100B and NSE have been reported in hypoglycemic children admitted to the pediatric intensive care unit (8). In addition, *in vitro* studies have shown that long-term glucose deprivation increases S100B and NSE release independently of hypoxia (32–34). In this study, hypoglycemic calves had significantly higher S100B and NSE concentrations than healthy calves (*p* < 00.5). Simultaneous elevation of S100B and NSE concentrations in hypoglycemic calves has been associated with the development of brain damage (8, 31–34). However, as S100B and NSE are not entirely brain specific, it should not be overlooked that they may also originate from peripheral tissues (35).

Astrocytes play a critical role in maintaining neuronal homeostasis in the brain by providing alternative fuel to neurons under hypoglycemic conditions (36, 37). Glial fibrillary acidic protein (GFAP), an intermediate cytoskeletal filament protein specific for astrocytes, is a key indicator of astrocyte activation. Expression of this protein outside the CNS is quite low, and the main causes of high serum concentrations are astrocyte activation after brain injury and regional necrosis (13). It has been reported that hypoglycemia-associated brain damage developed and GFAP release increased in rats experimentally induced with hypoglycemia (38). Similarly, an increase in the number of GFAP-positive astrocytes was found in rats with transient hypoglycemic coma (11). In contrast, GFAP expression was found to decrease in rat astrocytes with hyperglycemia (36, 37), and GFAP expression increased after correction of hyperglycemia (37). The authors suggest that astrocytes play a neuroprotective role during abnormal glucose homeostasis (36, 37). In the present study, GFAP concentrations were significantly higher in hypoglycemic calves than in healthy calves (*p* < 0.05). High GFAP concentrations have been associated with hypoglycemia-induced glial damage, astrocyte activation (11, 13) and neuroprotective role (36, 37).

Ubiquitin C-terminal hydrolase-1 (UCHL-1) is a highly abundant protein in neurons and neuroendocrine cells, constituting up to 5–10% of total neuronal proteins (14). Elevated concentrations in blood and cerebrospinal fluid (CSF) are associated with neuronal damage and increased permeability of the BBB (39). In a study of asphyxiated calves, elevated concentrations were associated with hypoxic–ischemic encephalopathy (12). It has also been reported to play a neuroprotective role in the repair process of damaged axons and neurons (40). In the present study, UCHL-1 concentrations in hypoglycemic calves were significantly higher than in healthy calves (*p* < 0.05). The high UHCL-1 concentrations in hypoglycemic calves may be due to neuroprotective properties rather than neuronal damage. In addition, it should be considered that neuroendocrine cells may be responsible for high UHCL-1 concentrations in hypoglycemic calves (14).

Activin A (ACT) has been shown as a neuronal protector in many CNS disorders (15, 41). *In vitro* and clinical studies have reported that high glucose concentrations increase ACT release (42–44). In contrast, ACT expression has been found to decrease after oxygen–glucose deprivation (10). Investigators (10, 42–44) have suggested a protective role for ACTs against the deleterious effects of inflammation, oxidative stress, and glucose dysregulation. In calves with perinatal asphyxia, low ACT concentrations were associated with species difference, oxidative stress, and overuse due to its role in repair (12). In the present study, serum ACT concentrations were found to be lower in hypoglycemic calves compared to healthy calves (*p* < 0.05). Low ACT concentrations in hypoglycemic calves may be associated with oxidative stress and neuroprotective properties (10, 12, 42, 43).

In hypoxic ischemia and hypoglycemia, increased AM expression has been observed in central cortical neurons, endothelial and perivascular glial cells (9). Similarly, a significant increase in plasma AM concentrations has been reported in hyperglycemic infants (45). However, no significant change in circulating AM concentrations was observed in insulin-induced hypoglycemia (46). This has been attributed to factors such as AM being unstable, having a short half-life of 20 min, and binding to some proteins in the circulation (47, 48). On the other hand, it has been suggested that AM concentrations are not affected by plasma glucose concentrations (49, 50). In the present study, there was no statistically significant difference in AM concentrations and no correlation with glucose concentrations (*p* > 0.05). These results suggest that AM does not play a role in the regulatory hormonal response to hypoglycemia (46, 49).

CK isoenzyme BB (CK-BB) is found at high levels in the brain and its activity is increased in peripheral blood in brain injury (17, 51). CK-BB is expressed by astrocytic glial cells (16). Experimentally, CK-BB enzyme activity was found to increase during insulin-induced hypoglycemia and showed a positive correlation with insulin dose (17). The increase in CK-BB enzyme activity in hypoglycemic cases has been related to the role of CK in brain energy demand and ATP production (brain energy hemostasis) (16, 52, 53). In the present study, the serum CK-BB enzyme activity of hypoglycemic calves was higher than that of healthy calves (*p* < 0.05). The elevated CK-BB enzyme activity in hypoglycemic calves is thought to be a response to brain energy and ATP demands (16, 52, 53) due to increased glycolysis, oxidative phosphorylation and sympathetic activation as a result of hypoglycemia.

Acute hypoglycemia alters blood levels of brain-derived proteins due to brain damage and BBB dysfunction caused by endothelial dysfunction and increased oxidative stress (6, 13). In the present study, blood glucose concentrations correlated negatively with S100B, GFAP, UCHL-1 concentrations and CK-BB enzyme activity and positively with ACT concentration. The simultaneous increase or decrease in serum concentrations of brain damage biomarkers and the significant correlation of these biomarkers with blood glucose concentrations suggest that brain damage develops in hypoglycemic calves (S100B, NSE, GFAP) and neuroprotective mechanisms (GFAP, UCHL-1, ACT, CK-BB) are activated to prevent damage (13).

Histopathologically, severe degeneration of glial cells (astrocytes, oligodendrocytes) occurs as a result of hypoglycemia, whereas ischemic neuronal damage is rare (7). Assessing the cell types from which brain-derived proteins originate in the present study, it can be assumed that glial cell damage (S100B, GFAP, CK-BB) rather than neuronal damage (NSE, UCHL-1) occurs in hypoglycemic calves (7). However, histopathology studies are required to prove this in hypoglycemic calves.

In the present study, although there was a significant difference in brain injury biomarker concentrations between healthy and hypoglycemic calves, these biomarker concentrations were not good indicators for predicting mortality. Therefore, to determine the importance of other clinicopathologic variables on mortality, a logistic regression model was constructed. In the present study, the variables of severe hypoglycemia, severe acidosis, and sepsis were not significant in predicting prognosis when evaluated alone or together. Furthermore, when assessing model capacity, severe hypoglycemia, severe acidosis, and sepsis had low sensitivity and specificity in predicting mortality in hypoglycemic calves. These results are consistent with the view that laboratory parameters have limited value in predicting mortality, but the presence of specific clinical abnormalities provides valuable prognostic information (19). Based on our clinical experience, the survival of all calves within the first 12 h indicates that the duration of hypoglycemia could have a direct effect on mortality.

However, the study has some limitations, including (i) the absence of histopathologic evaluation of hypoglycemic calves for brain damage, (ii) the lack of measurement of CSF concentrations of the biomarkers used, and (iii) no inclusion of a group of non-hypoglycemic calves with diarrhea. All these aspects deserve to be addressed in further studies.

The results from the present study conclude that hypoglycemia-associated brain damage developed in hypoglycemic calves with diarrhea. This damage occurred in glial cell populations rather than neurons and caused changes in serum concentrations of brain biomarkers. In addition, hypoglycemia increased mortality, but biomarkers of brain injury were not useful in predicting mortality due to low sensitivity and specificity, and severe hypoglycemia, severe acidosis, and sepsis variables alone or together were not effective in predicting mortality.

## Data availability statement

The raw data supporting the conclusions of this article will be made available by the authors, without undue reservation.

## Ethics statement

The animal study was reviewed and approved by Ethics Committee of the Faculty of Veterinary Medicine, Selcuk University. The studies were conducted in accordance with the local legislation and institutional requirements. Written informed consent was obtained from the owners for the participation of their animals in this study.

## Author contributions

MI and AN: writing—original draft preparation, conceptualization, and methodology. MI and AE: investigation. SSI and MKD: data curation. MO: writing—review and editing. All authors contributed to the article and approved the submitted version.
